# Analgesia and Pain in Female and Male Patients After Video-Assisted Thoracic Surgery: A Study Under Real-World Conditions

**DOI:** 10.3390/jcm15041397

**Published:** 2026-02-10

**Authors:** Bernhard Zapletal, Patricia Schukro, Thomas Schweiger, Merjem Begic, Edda M. Tschernko

**Affiliations:** 1 Department of Anesthesiology, General Intensive Care and Pain Medicine, Division of Cardiac Thoracic Vascular Anesthesia and Intensive Care Medicine, Medical University Vienna, Waehringer Guertel 18–20, 1090 Vienna, Austria; regina.schukro@muv.ac.at (P.S.); edda.tschernko@meduniwien.ac.at (E.M.T.); 2 Department of Thoracic Surgery, Medical University Vienna, Waehringer Guertel 18–20, 1090 Vienna, Austriamerjem.begic@muv.ac.at (M.B.)

**Keywords:** female sex, male sex, pain therapy, thoracic surgery, VATS

## Abstract

**Background**: Mounting evidence suggests that medical management may differ significantly between female and male patients. Despite studies showing increased sensitivity to pain, female patients receive less opioid analgesia compared to male patients after surgery. It is uncertain whether perioperative multimodal analgesia differs between sexes in thoracic surgery. **Methods**: A retrospective cohort study from January to July 2023 comparing multimodal analgesia and perceived pain in the early postoperative period between female and male patients after video-assisted thoracic surgery (VATS). The primary endpoint was the opioid demand in the post-anaesthesia care unit (PACU). Secondary outcomes included pain scores, regional anaesthesia and pain therapy by female, male or mixed teams. **Results**: Overall, 46.0% (*n* = 92) of the 200 included patients were female and 54% (*n* = 108) were male. Following VATS, the median piritramide demand was 9.0 [5.3 to 14.9] mg in female vs. 7.7 [4.5 to 12.9] mg in male patients (*p* = 0.35). Pain scores and regional anaesthesia were comparable between groups. In the early postoperative period, more opioids were administered overall and to female patients by all female anaesthesia teams, compared to mixed or all-male teams. **Conclusions**: The weight-adjusted dose of postoperative opioids did not differ between groups; neither did postoperative pain scores or the application of nerve blocks. The increased opioid demand in female patients was met by all female teams but not by all-male or mixed teams.

## 1. Introduction

Sex-based differences exist regarding the clinical presentation and treatment of various diseases [1,2,3,4,5,6]. Regarding postoperative pain, studies after video-assisted thoracic surgery (VATS) and colorectal surgery have shown increased postoperative pain scores in female patients, while the postoperative opioid dose did not differ significantly between female and male patients, indicating insufficient pain therapy in female patients [7,8]. By contrast, studies after cardiac surgery have shown no difference in postoperative pain scores and significantly fewer postoperative opioids administered in female patients compared to male patients [9,10]. Large reviews including thoracic or cardiac surgical procedures show female patients to be particularly at risk for increased postoperative pain compared to male patients [11]. Differences in central pain processing, particularly reduced endogenous pain inhibition, may explain the greater postoperative pain in women [11,12,13]. This is quite alarming since it may be interpreted as female patients receiving fewer opioids than male patients, despite comparable or increased pain, which may be the result of prescriber bias, women reporting less severe postoperative pain to male assessors or fear of opioid-associated side effects in women [14,15,16]. 

This discrepancy is relevant since inadequate analgesia may impair quality of life and might slow down recovery after surgery by preventing the adequate clearance of secretions and inducing atelectasis, with an increased risk of postoperative pneumonia—a risk that is particularly pronounced in the immediate postoperative period [17,18,19]. Early postoperative pain has also been associated with prolonged lengths of stay in the post-anaesthesia care unit (PACU), therefore increasing resource utilisation [20,21,22]. Additionally, insufficient control of early postoperative pain may increase myocardial stress and promote the development of chronic postoperative pain [23,24,25,26].

We investigated differences in early postoperative opioid administration and postoperative pain after video-assisted thoracic surgery (VATS). Based on existing data, we hypothesised that postoperative pain and opioid doses would be different between male and female patients. We aimed to evaluate sex differences in postoperative pain perception and pain after VATS in a real-world therapeutic setting.

## 2. Methods

### 2.1. Design

This was a retrospective single-centre study performed in patients undergoing VATS from January to July 2023 in a tertiary hospital. The study protocol was approved by the Institutional Review Board (EC number 2204/2023).

### 2.2. Patients

We included 200 consecutive patients (i) aged 18 years or older, (ii) who underwent VATS, (iii) and who were admitted to the PACU after surgery. We excluded patients who received regional anaesthesia other than an erector spinae plane block (ESPB) or intercostal block (ICB) or received opioids other than piritramide postoperatively. We also excluded patients with preoperative opioid use or with chronic pain conditions before surgery. Patients with postoperative prolonged intubation or treatment in an ICU instead of the PACU after surgery, and patients with missing information about pain management, were also excluded.

### 2.3. Intravenous Combination Analgesia

At our centre, during general anaesthesia, remifentanil and fentanyl are combined with propofol and rocuronium. In patients managed with remifentanil, a bolus (2 to 3 µg.kg^–1^ body weight) of fentanyl is administered at the induction of anaesthesia and another (1 to 2 µg.kg^–1^ body weight) at the end of the remifentanil infusion, with no other long-acting opioids used intraoperatively. Two of the non-opioids metamizole (G.L. Pharma, Lannach, Austria), acetaminophen (Industria Farmaceutica Galenica, Monteroni D’arbia, Italy), and diclofenac (Fresenius Kabi, Seiersberg, Austria) are combined with piritramide (HBM Pharma, Martin, Slovakia) in weight-adjusted doses (1.5 to 4.5 mg i.v.) if the visual analogue scale (VAS) score exceeds 3 in the PACU.

### 2.4. Erector Spinae Plane Block and Intercostal Block

At our centre, the erector spinae plane block (ESPB) is performed under general anaesthesia in the lateral decubitus position immediately after skin closure. After locating the transverse process T4–6 and achieving good visual condition of the superficial muscles using a 13–6 MHz linear array US transducer (HFL38xi, Sonosite, Bothell, WA, USA) in the cephalad-to-caudal direction, a 70 mm 22-gauge needle (USC 70 Evolution, Felsberg, Germany) is inserted using an in-plane approach. After confirming optimal needle tip position by hydrodissection, an interfascial injection of 30 mL 0.375% ropivacaine (Sintetica, Mendrisio, Switzerland) combined with 0.5–1 µg.kg^–1^ of dexmedetomidine (Orion Pharma Austria, Vienna, Austria) was performed, with dose reduction to 20 mL 0.375% ropivacaine in patients below 50 kg BW.

The intercostal block (ICB) was performed intraoperatively by the thoracic surgery team under single-lung ventilation. Before the end of the surgical procedure, a total of 15 to 20 mL 0.75% ropivacaine (4 mL per intercostal space) was injected 2 to 4 cm laterally of the costotransverse joint—typically two intercostal spaces above and two below the thoracoscopic incision site. Correct needle placement was confirmed by thoracoscopic visualisation via the identification of the intercostal nerve and pleural detachment following the injection. The ropivacaine dose was reduced to 15 mL in patients below 50 kg BW.

### 2.5. Data Collected

Demographic data and baseline characteristics for each patient, including their sex, age, American Society of Anesthesiology (ASA) score, and primary diagnosis, were collected at baseline. The sexes of the anaesthesia consultant and resident, as well as the PACU nurses, were derived from the medical records. Additionally, the postoperative piritramide demand, the type and dosage of intraoperative opioids, the intra- and postoperative non-opioid analgesics, and the use of regional anaesthesia (ESPB and ICB) were evaluated for all patients. Pain levels were assessed routinely every 30 min in the PACU by nursing staff using the VAS score from 0 to 10 [17], and sedation levels were assessed using the modified Ramsay sedation scale (mRSS) [18] until discharge to the ward. Patients with missing primary outcome data were excluded from the analysis after screening.

### 2.6. Definitions

Operating time encompassed the duration of surgery from skin incision to skin closure. All data were extracted from our automated patient data management system. Anaesthesia teams consisted of the anaesthesia resident and the anaesthesia consultant in charge of the case, as well as the anaesthesia nurse being in charge of postoperative treatment in the PACU. All female anaesthesia teams were defined as teams in which the anaesthesia resident and consultant, as well as the PACU nurse, were of the female sex. All male anaesthesia teams were defined as teams in which the anaesthesia resident and consultant, as well as the PACU nurse, were of the male sex. Mixed teams were defined as consisting of male and female anaesthesia providers and PACU nurses. The influence of the operating surgeon’s sex was not investigated.

### 2.7. Study Endpoints

The primary endpoint was the cumulative postoperative piritramide demand during the first 2 h after arrival in the PACU. In our centre, piritramide is the standard intravenous medication for postoperative analgesia in the PACU following thoracic surgery due to its moderately long duration of action, broad therapeutic range, and familiarity among all local caregivers [27]. The therapeutic goal for postoperative pain therapy after thoracic surgery is to achieve a VAS score < 3 by titrating piritramide until this goal is reached. Therefore, we chose the amount of piritramide as the primary outcome measure.

As a secondary outcome, we aimed to evaluate the influence of the analgesia-providing team’s sex on the amount of piritramide administered according to the sex of the patient. The secondary endpoints included pain scores (VAS) and sedation scores (mRSS) in the PACU, intraoperative opioids used, and intra- and postoperative non-opioids administered. We also report the operating time, the length of stay (LOS) in the PACU, the postoperative LOS in the hospital, and adverse events. We chose to compare the postoperative piritramide demand and VAS for patients with LOS > 6 h in the PACU during that time period.

### 2.8. Sample Size Calculation

Based on the results of previous works showing that female patients received 31% fewer opioids compared to male patients [9], and previously published data from our own centre that showed a mean early postoperative piritramide demand of 9.0 (6.4) mg in all patients [28], we hypothesised that female patients would receive 7.6 mg piritramide in the early postoperative period, compared to 10.4 mg in male patients. We estimated that a sample of 83 patients per group (a total of 166) would provide the trial with 80% power to detect a clinically significant difference in the postoperative piritramide demand between female and male patients at a two-sided alpha level of 0.05. To account for the unequal sample sizes due to slightly fewer female patients undergoing VATS at our centre compared to male patients [28], and to account for dropouts, the sample was increased by 25% to a total of 208 consecutive patients.

### 2.9. Statistical Analysis

We compared the postoperative piritramide demand in female and male patients after VATS. The variables are presented using counts and percentages, means and standard deviations (SDs), or medians and interquartile ranges (IQRs), depending on the data distribution as assessed using the Shapiro–Wilk test and inspection of histograms.

To evaluate the primary and secondary outcomes, we employed a two-sided superiority hypothesis. The mean overall and weight-adjusted differences in the postoperative piritramide demand comparing female and male patients, the use of regional anaesthesia, and differences regarding the postoperative piritramide dose in all-female, all-male, and mixed anaesthesia teams are reported.

All statistical analyses were performed using SPSS for Windows, version 29.0 (IBM Corp., Armonk, NY, USA), and GraphPad Prism for Windows, version 10.0.0 (GraphPad Software, Boston, MA, USA). We considered a *p*-value of less than 0.05 as statistically significant.

## 3. Results

### 3.1. Patients

**Figure 1:**
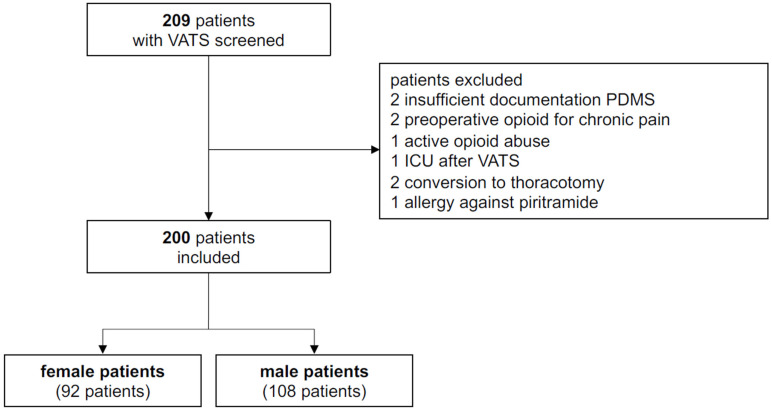
Flowchart of patient inclusion process. Abbreviations: VATS, video-assisted thoracic surgery; PDMS, patient data management system; ICU, intensive care unit.

From January to July 2023, 208 patients underwent VATS. The main reasons for exclusion from the analysis were insufficient documentation of VAS or analgesia, preoperative opioid use, and conversion to thoracotomy (Figure 1). Of the included 200 patients, 46.0% (*n* = 92) were female and 54% (*n* = 108) were male. The majority of patients had a malignancy and underwent elective surgery. Urgent surgery was more frequent in male patients (Table 1). Diagnoses and surgical procedures were comparable between female and male patients. Diagnostic VATS and VATS–pleurectomy were performed more frequently in male patients (Table 1).

### 3.2. Postoperative Piritramide

**Figure 2:**
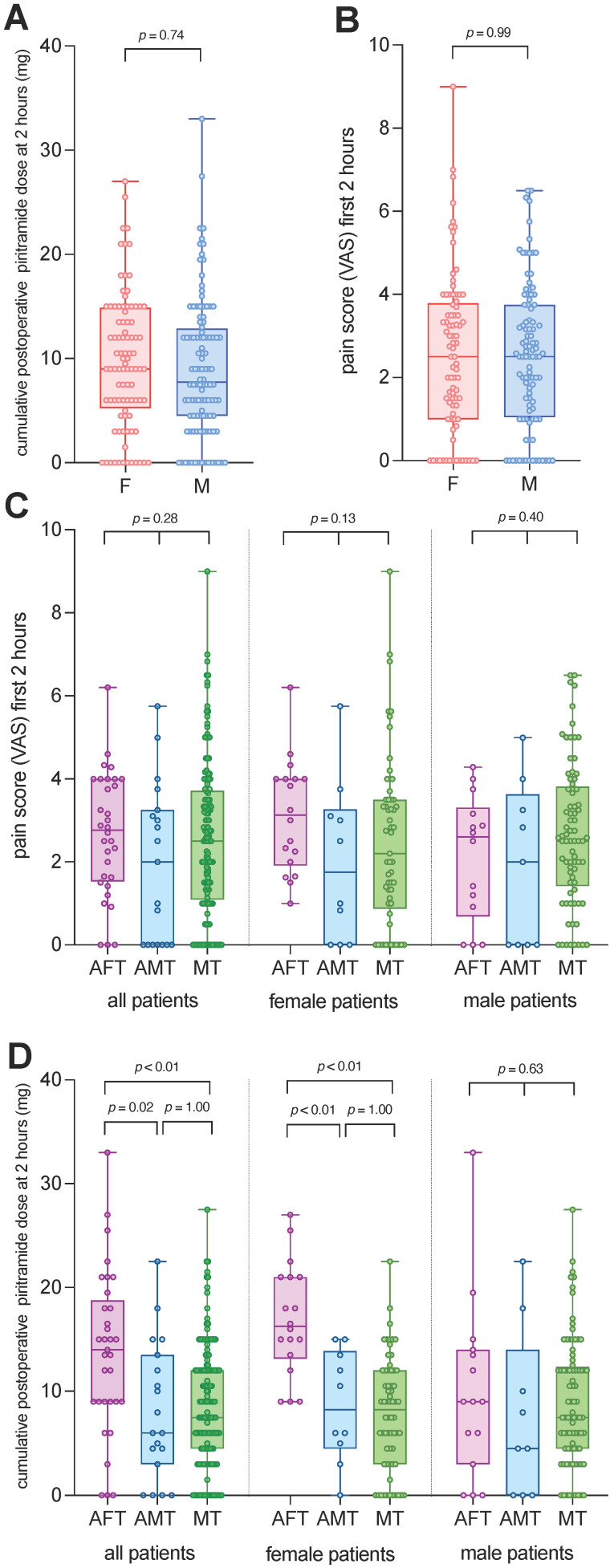
Box and whisker plots of postoperative piritramide dose in female and male patients and pain scores. (**A**) Piritramide dose and (**B**) pain scores in patients after VATS; (**C**) pain scores in all patients, female patients, and male patients treated by all-female, all-male, and mixed anaesthesia teams; (**D**) piritramide dose in all patients, female patients, and male patients treated by all-female, all-male, and mixed anaesthesia teams. Abbreviations: F, female patients; M, male patients; VAS, visual analogue scale; AFT, all-female anaesthesia team; AMT, all-male anaesthesia team; MT, mixed anaesthesia team.

The postoperative piritramide demand in the PACU within 2 h of arrival in the PACU was 9.0 [5.3 to 14.9] mg in female patients, compared to 7.7 [4.5 to 12.9] mg in male patients (*p* = 0.35, Figure 2A and Table 2). The weight-adjusted piritramide demand in the PACU did not differ between female and male patients (*p* = 0.928, Table 2).

### 3.3. Postoperative Pain Scores

The VAS during the first 2 h in the PACU did not differ between female and male patients (Figure 2B and Table 2).

### 3.4. Influence of Treatment Team’s Sex

The VAS during the first 2 h in the PACU did not differ according to whether patients were treated by all-female, all-male or mixed teams or whether patients were male or female. Overall, significantly more piritramide was administered by all-female teams (14.0 [9.0 to 18.8] mg) compared to all-male (6.0 [3.0 to 13.5] mg, *p* = 0.02) and mixed teams (7.5 [4.5 to 12.0] mg, *p* ≤ 0.01). Significantly more piritramide was also administered by all-female teams to female patients (16.3 [13.1 to 21.0] mg) compared to all-male teams (8.3 [4.5 to 13.9] mg, *p* < 0.01) and mixed teams (8.3 [3.0 to 12.0] mg, *p* < 0.01) (Figure 2C,D and Table 3). Adverse events including PONV and LOS in the PACU did not differ between patients treated by all-female, all-male and mixed anaesthesia teams (Supplementary Materials, Table S1).

### 3.5. Additional Endpoints

There were no differences regarding the use of ICB and ESPB, sedation scores in the PACU, regional anaesthesia, LOS in the PACU, LOS in the hospital, in-hospital mortality, or perioperative non-opioid analgesia between female and male patients. The postoperative piritramide demand and VAS during the first 6 h in patients with LOS > 6 h in the PACU did not differ between female and male patients (*p* = 0.88 and *p* = 0.65, Table S2). Remifentanil was more frequently used in male (*n* = 103 (95.4%)) compared to female patients (*n* = 78 (84.8%), *p* = 0.02) (Table 2).

## 4. Conclusions

Our findings can be summarised as follows: (1) the early postoperative opioid demand in the PACU did not differ between female and male patients after VATS; (2) neither did early postoperative pain scores (3) or the use of regional anaesthesia; (4) more piritramide was administered by all-female anaesthesia teams overall (4) and by all female teams to female patients compared to all-male or mixed anaesthesia teams; and (5) this was not associated with differences in any adverse events, including sedation and PONV, and did not increase the LOS in the PACU.

To the best of our knowledge, this is the first investigation comparing postoperative analgesia and pain in female and male patients after VATS and assessing the potential roles of all-female, all-male and mixed anaesthesia teams in perioperative pain therapy. Consecutive patients were included until the previously calculated sample size was reached, with exclusion criteria limited to patients under preoperative opioids or remaining intubated. The prespecified analysis plan was strictly adhered to after the dataset was examined for completeness. Standardised pain therapy aiming for a VAS < 3 and the evaluation of pain and sedation scores is routinely performed and documented by the nursing team in the PACU; thus, our study reflected a real-world treatment scenario. Due to the retrospective nature of the study, the anaesthesiologists and nurses were unaware of the study and not prone to documentation or treatment bias.

Numerous investigations in various acute conditions have shown inferior outcomes or suboptimal treatment in female patients compared to males, resulting in delayed assessment, delayed treatment, increased mortality, lower odds of being prescribed opioid analgesics, and being treated with benzodiazepines rather than analgesics for pain. Overall, female patients are often at a structural disadvantage in the medical system [29,30,31].

Several investigations and meta-analyses have examined postoperative pain in female and male patients and healthy volunteers. Overall, painful stimuli or postoperative pain were perceived as more intense by female volunteers [32,33], and the female sex was found to negatively influence postoperative pain in the majority of surgical procedures [11]. Hrebinko and colleagues examined pain control after colorectal surgery and observed no significant difference regarding the dose of opioids administered postoperatively, despite increased postoperative pain scores in female patients [7]. Karamesinis et al. observed that female patients received significantly fewer opioids after cardiac surgery, which may indicate a reduced opioid demand or insufficient pain therapy in females [9]. Undertreatment may occur through physician bias, falsely attributing female patients’ symptoms and complaints to anxiety or emotional discomfort rather than recognising and treating them as pain [15,34]. Patient–physician sex discordance, with female patients reporting lower pain scores for male than for female investigators, might also impair postoperative pain therapy in women [16]. A pioneering investigation in this field reported that fewer opioids and more sedatives were administered for pain complaints in female patients compared to male patients [35]. Thus, data regarding the influence of the patient’s sex on pain therapy are conflicting and lacking, particularly after thoracic surgery. In our investigation, the perceived levels of pain and administered opioids were similar between sexes during the early postoperative period. This can be explained by the adherence to a strict protocol evaluating pain and sedation scores in all patients during the initial postoperative period, with a minimum interval of 30 min or at a higher frequency.

Regional anaesthesia by ESPB or ICB was performed at a high (>70% of all patients) and equal rate in female and male patients for multimodal analgesia. This practice could certainly influence pain control in the early postoperative period and should be advocated for in all patients [25]. Teams of all-female caregivers administered significantly more piritramide overall compared to all-male and mixed teams. Teams of all-female caregivers also administered significantly more piritramide to female patients than all-male and mixed teams. No such effect was found in male patients.

Our data are in line with mounting evidence from large collectives of surgical and medical patients alike, indicating clear differences in treatment due to the caregiver’s sex. This effect was found to be particularly pronounced in female patients receiving treatment by female physicians [36,37,38]. The underlying cause cannot be determined with certainty, leaving room for speculation. The opioid dose delivered via patient-controlled analgesia (PCA) is the gold standard in intravenous opioid analgesia and might rule out potential sex differences in postoperative pain therapy [39,40,41]. No adverse effects of the increased opioid dose provided to female patients by all-female teams of caregivers were observed in our study. Undesirable opioid-associated side effects include PONV and oversedation, leading to an increased LOS in the PACU and impairing early recovery after surgery; these were not observed in our study [23,25,42,43].

Our study has limitations—mainly the relatively short 2 h observation period for the primary outcome and the retrospective design. The observation period was chosen for several reasons. Most patients are transferred to the ward after two hours if postoperative recovery is uneventful. Furthermore, pain monitoring and analgesia are more standardised in our PACU than after transfer to the surgical ward. While 2 h is a short period, the reduction of early postsurgical pain is essential to reduce myocardial stress and improve effective coughing while reducing opioid-associated side effects, as well as preventing the development of chronic postoperative pain caused by inadequate analgesia [23,25]. Of note, the results in the 6 h observation period were consistent with the findings of the 2 h analysis. The number of female and male patients treated by all-female and all-male teams was relatively small compared to the number of patients treated by mixed teams. Therefore, we cannot exclude the possibility that the observed effects arose by chance. Due to the study design, we also cannot exclude the influences of other factors, including patients’ comorbidities or the influence of specific surgeons or anaesthesiologists on the results. Demographic differences may also have affected the results, including more urgent surgery and a higher percentage of male patients receiving remifentanil. Urgent surgery is unlikely to have influenced the results, as such procedures at our centre are performed within 24 h and differ only marginally from elective surgery. While intraoperative fentanyl was used in the overwhelming majority of patients, remifentanil was used in a higher proportion of male patients. Although remifentanil is usually not used in doses above 0.25 µg/KG BW/min in our centre, and the intraoperative weight-adjusted dose did not differ between female and male patients, potentially occurring hyperalgesia associated with remifentanil might therefore have affected more male compared to female patients [35,36]. The PACU nurses were not blinded, but, since this was a retrospective analysis, they were unaware of the study. The VAS at deep inspiration or cough could not be compared between groups, since the VAS is measured and documented at rest only in our PACU.

Based on these results, we conclude that the early opioid demand and early postoperative pain after VATS were comparable between female and male patients, with no differences in undesirable side effects. The aim of pain therapy, i.e., to achieve a VAS < 3, was achieved in both sexes, underscoring the effectiveness of a stringent postoperative protocol. The observation that patients received more opioids if they were cared for by all-female teams has to be noted with caution due to the small number of patients examined and the fact that adequate pain control could be achieved by all groups of caregivers alike. However, increased opioid administration was not associated with an increase in opioid-related adverse effects. The effect of the sex difference in caregivers regarding pain therapy is to be regarded as a signal at this point and should be further explored in the future.

## Figures and Tables

**Table 1:** Patient demographics and baseline characteristics.

	Female Patients (*n* = 92)	Male Patients (*n* = 108)	*p*
Age, years	65.1 [55.6 to 73.7]	63.2 [47.9 to 72.9]	0.23
Weight, kg	68.0 [60.0 to 80.0]	78.0 [66.0 to 91.8]	<0.01
BMI, mean (SD)	25.0 [21.3 to 29.7]	25.2 [22.4 to 27.9]	0.79
ASA score	3.0 [2.0 to 3.0]	3.0 [2.0 to 3.0]	0.68
Type of surgery			
Elective surgery, *n* (%)	91 (98.9)	97 (89.8)	0.03
Urgent surgery, *n* (%)	1 (1.1)	11 (11.2)	
Regional anaesthesia			
ESPB, *n* (%)	46 (50.0)	55 (50.9)	0.90
ICB, *n* (%)	20 (21.7)	16 (14.8)	0.20
No RA, *n* (%)	26 (28.3)	37 (34.3)	0.36
Primary diagnosis			0.14
Malignancy, *n* (%)	67 (72.8)	64 (59.2)	
Pathology pending	42	35 (32.4)	
Adenocarcinoma	12	12 (11.1)	
Squamous cell carcinoma	4	6 (5.6)	
Non-small-cell lung cancer	3	3 (2.8)	
Metastasis	5	5 (4.6)	
Other malignancy	1	3 (2.8)	
Pneumothorax, *n* (%)	2 (2.2)	19 (17.6)	
Pleural effusion, *n* (%)	9 (9.8)	9 (8.3)	
Haemothorax, *n* (%)	2 (2.2)	2 (1.9)	
Empyema, *n* (%)	4 (4.3)	5 (4.6)	
Emphysema, *n* (%)	1 (1.1)	2 (1.9)	
Other, *n* (%)	7 (7.6)	7 (6.5)	
Surgical intervention			0.06
EASR/ASR, *n* (%)	39 (42.4)	35 (32.4)	
Single EASR, *n*	20	20	
EASR + ASR, *n*	1	0	
Multiple EASR, *n*	10	8	
Multiple EASR + ASR, *n*	1	0	
Single ASR, *n*	5	5	
Multiple ASR, *n*	1	2	
ASR + diaphragm reconstruction	1	0	
Lobectomy/pneumonectomy, *n* (%)	25 (27.2)	24 (22.2)	
Lobectomy, *n*	20	19	
Lobectomy + EASR, *n*	5	4	
Bilobectomy, *n*	0	1	
Pleurodesis	7 (7.6)	3 (2.8)	
Pleurodesis	6	3	
Pleurodesis + IPC	1	0	
Diagnostic VATS, *n* (%)	7 (7.6)	13 (12.0)	
Diagnostic VATS	1	10	
Diagnostic VATS + IPC	3	2	
Diagnostic VATS + pleurectomy	1	0	
Diagnostic VATS + single EASR	2	0	
Diagnostic VATS + multiple EASR	0	1	
Pleurectomy	6 (6.5)	24 (22.2)	
Pleurectomy	5	17	
Pleurectomy + EASR	1	7	
Miscellaneous, *n* (%)	9 (9.8)	8 (7.4)	
Bullectomy	0	1	
Haematoma evacuation	1	0	
Other	8	7	

Data are presented as mean (SD) or as numbers (%). Abbreviations: BMI, body mass index; ASA, American Society of Anesthesiologists Physical Status Classification; ESPB, erector spinae plane block; ICB, intercostal block; RA, regional anaesthesia; EASR, extraanatomical segment resection; ASR, anatomical segment resection; IPC, intrapleural catheter; VATS, video-assisted thoracic surgery.

**Table 2:** Piritramide dose and VAS for pain in the first 2 h after arrival in the PACU.

	Female Patients (*n* = 92)	Male Patients (*n* = 108)	*p*
Primary outcome			
Median piritramide dose in first 2 h, mg [IQR]	9.0 [5.3 to 14.9]	7.7 [4.5 to 12.9]	0.35
Median piritramide dose per kg BW in first 2 h, mg [IQR]	0.14 [0.07–0.21]	0.10 [0.05–0.18]	0.09
Secondary outcomes			
Median VAS for pain in first 2 h [IQR]	2.5 [1.0 to 3.8]	2.5 [1.0 to 3.8]	0.99
Sedation score in first 2 h, mg, median [IQR]	3.0 [2.3 to 3.0]	3.0 [2.5 to 3.0]	0.45
Operating time, min, median [IQR]	95.5 [57.0 to 155.5]	91.5 [49.8 to 139.8]	0.14
LOS			
In PACU, min, median [IQR]	120.0 [105.0 to 163.8]	130.0 [108.3 to 180.0]	0.41
In hospital, days, median [IQR]	4.0 [2.8 to 5.0]	3.0 [3.0 to 5.0]	0.59
Death in hospital	0 (0.0)	2 (1.9)	0.50
Analgesia			
Intraoperative:			
Remifentanil, *n* (%)	78 (84.8)	103 (95.4)	0.02
Remifentanil maintenance dose, µg per kg BW per minute	0.23 [0.21 to 0.28]	0.24 [0.21 to 0.26]	0.54
Fentanyl, *n* (%)	74 (80.4)	86 (79.6)	0.89
Fentanyl, µg, median [IQR]	275.0 [150.0 to 350.0]	275.0 [150.0 to 168.8]	0.74
Intra- and postoperative:			
Metamizole, *n* (%)	80 (87.0)	92 (85.2)	0.84
Metamizole, mg, median [IQR]	1000 [1000.0 to 2500.0]	2000.0 [1000.0 to 2500.0]	0.39
Diclofenac, *n* (%)	50 (54.3)	52 (48.1)	0.40
Diclofenac (mg)	75.0 [0.0 to 75.0]	0.0 [0.0 to 75.0]	0.39
Acetaminophen, *n* (%)	42 (45.7)	42 (38.9)	0.40
Acetaminophen, mg, median [IQR]	0.0 [0.0 to 1000.0]	0.0 [0.0 to 1000.0]	0.31

Data are presented as median with interquartile range [IQR]. Abbreviations: PACU, post-anaesthesia care unit; BW, body weight; VAS, visual analogue scale; LOS, length of stay. VAS used a scale of 1 to 10; sedation score used a scale between 0 and 3.

**Table 3:** Piritramide dose and VAS in PACU according to sex of treatment team.

	All-Female Teams (AFT, *n* = 33)	All-Male Teams (AMT, *n* = 19)	Mixed Teams (MT, *n* = 148)	*p*
All patients				
Median VAS for pain in first 2 h [IQR]	2.8 [1.5 to 4.0]	2.0 [0.0 to 3.3]	2.5 [1.1 to 3.7]	0.28
Median piritramide dose in first 2 h, mg [IQR]	14.0 [9.0 to 18.8]	6.0 [3.0 to 13.5]	7.5 [4.5 to 12.0]	AFT-AMT 0.02 AFT-MT < 0.01 AMT-MT 1.0
Median piritramide dose per kg BW in first 2 h, mg [IQR]	0.2 [0.1 to 0.3]	0.1 [0.0 to 0.2]	0.1 [0.1 to 0.2]	AFT-AMT 0.03 AFT-MT < 0.01 AMT-MT 1.0
Female patients				
Median VAS for pain in first 2 h [IQR]	3.1 [1.9 to 4.0]	1.8 [0.0 to 3.2]	2.2 [0.9 to 3.5]	0.13
Median piritramide dose in first 2 h, mg [IQR]	16.3 [13.1 to 21.0]	8.3 [4.5 to 13.9]	8.3 [3.0 to 12.0]	AFT-AMT < 0.01 AFT-MT < 0.01 AMT-MT 1.0
Median piritramide dose per kg BW in first 2 h, mg [IQR]	0.3 [0.2 to 0.3]	0.1 [0.1 to 0.2]	0.1 [0.1 to 0.2]	AFT-AMT = 0.01 AFT-MT < 0.01 AMT-MT 1.0
Male patients				
Median VAS for pain in first 2 h [IQR]	2.6 [0.7 to 3.3]	2.0 [0.0 to 3.6]	2.5 [1.4 to 3.8]	0.40
Median piritramide dose in first 2 h, mg [IQR]	9.0 [3.0 to 14.0]	4.5 [0.0 to 14.0]	7.5 [4.5 to 12.4]	0.63
Median piritramide dose per kg BW in first 2 h, mg [IQR]	0.1 [0.0 to 0.2]	0.1 [0.0 to 0.2]	0.1 [0.1 to 0.2]	0.66

Data are presented as median with interquartile range [IQR]. Abbreviations: PACU, post-anaesthesia care unit; BW, body weight; VAS, visual analogue scale. VAS used a scale of 1 to 10.

## Data Availability

The dataset for the current study is available on request via the corresponding author.

## Supplementary Materials

The following supporting information can be downloaded at https://www.mdpi.com/article/10.3390/jcm15041397/s1. Table S1. Adverse events and LOS in the PACU. Table S2. VAS and piritramide dose in first 6 h after arrival in the PACU. Figure S1. VAS and piritramide dose in first 6 h after arrival in the PACU.

## References

[1] Manzo-Silberman S., Couturaud F., Charpentier S., Auffret V., Khoury C.E., Breton H.L., Belle L., Marlière S., Zeller M., Cottin Y. (2018). Influence of Gender on Delays and Early Mortality in ST-Segment Elevation Myocardial Infarction: Insight from the First French Metaregistry, 2005–2012 Patient-Level Pooled Analysis. Int. J. Cardiol..

[2] Zhao M., Woodward M., Vaartjes I., Millett E.R.C., Klipstein-Grobusch K., Hyun K., Carcel C., Peters S.A.E. (2019). Sex Differences in Cardiovascular Medication Prescription in Primary Care: A Systematic Review and Meta-Analysis. J. Am. Heart Assoc..

[3] May L., Shows K., Nana-Sinkam P., Li H., Landry J.W. (2023). Sex Differences in Lung Cancer. Cancers.

[4] Lusk J.B., Ford C.B., Soneji S., Blass B., Rosa T.D., Kaufman B.G., Mantri S., Li F., Grory B.M., Xian Y. (2025). Sex Differences in Mortality and Health Care Utilization After Dementia Diagnosis. JAMA Neurol..

[5] Coventry L.L., Finn J., Bremner A.P. (2011). Sex Differences in Symptom Presentation in Acute Myocardial Infarction: A Systematic Review and Meta-Analysis. Heart Lung J. Acute Crit. Care.

[6] Abusnina W., Elhouderi E., Walters R.W., Al-Abdouh A., Mostafa M.R., Liu J.L., Mazozy R., Mhanna M., Ben-Dor I., Dufani J. (2024). Sex Differences in the Clinical Outcomes of Patients With Takotsubo Stress Cardiomyopathy: A Meta-Analysis of Observational Studies. Am. J. Cardiol..

[7] Hrebinko K.A., Myers S.P., Tsang W.L., Doney L., Lazar S., Teng C., Subramaniam K., Holder-Murray J. (2020). Sex Comparisons in Opioid Use and Pain After Colorectal Surgery Using Enhanced Recovery Protocols. J. Surg. Res..

[8] Wang H., Luo M., Yang Y., Li S., Liang S., Xu R., Zhu J., Song B. (2024). Gender Differences in Postoperative Pain, Sleep Quality, and Recovery Outcomes in Patients Undergoing Visual Thoracoscopic Surgery. Heliyon.

[9] Karamesinis A.D., Neto A.S., Shi J., Fletcher C., Hinton J., Xing Z., Penny-Dimri J.C., Ramson D., Liu Z., Plummer M. (2024). Sex Differences in Opioid Administration After Cardiac Surgery. J. Cardiothorac. Vasc. Anesth..

[10] Perry L.A., Brooks G.G., Greifer N., Hua J., Phillips B., Silvers A., Yang B.O., Tsigaras Z., Borann B., Hui V. (2025). The Influence of Sex on Early Postoperative Opioid Administration after Cardiac Surgery. Aust. Crit. Care.

[11] Pereira M.P., Pogatzki-Zahn E. (2015). Gender Aspects in Postoperative Pain. Curr. Opin. Anaesthesiol..

[12] Popescu A., LeResche L., Truelove E.L., Drangsholt M.T. (2010). Gender Differences in Pain Modulation by Diffuse Noxious Inhibitory Controls: A Systematic Review. Pain.

[13] Fillingim R.B., Maixner W., Kincaid S., Silva S. (1998). Sex Differences in Temporal Summation but Not Sensory-Discriminative Processing of Thermal Pain. Pain.

[14] Dewinter G., Staelens W., Veef E., Teunkens A., de Velde M.V., Rex S. (2018). Simplified Algorithm for the Prevention of Postoperative Nausea and Vomiting: A before-and-after Study. Br. J. Anaesth..

[15] Hoffmann D.E., Tarzian A.J. (2001). The Girl Who Cried Pain: A Bias against Women in the Treatment of Pain. J. Law Med. Ethics.

[16] Meyer-Frießem C.H., Szalaty P., Zahn P.K., Pogatzki-Zahn E.M. (2019). A Prospective Study of Patients’ Pain Intensity after Cardiac Surgery and a Qualitative Review: Effects of Examiners’ Gender on Patient Reporting. Scand. J. Pain.

[17] Shea R.A., Brooks J.A., Dayhoff N.E., Keck J. (2002). Pain Intensity and Postoperative Pulmonary Complications among the Elderly after Abdominal Surgery. Heart Lung J. Acute Crit. Care.

[18] Pöpping D.M., Elia N., Marret E., Remy C., Tramèr M.R. (2008). Protective Effects of Epidural Analgesia on Pulmonary Complications After Abdominal and Thoracic Surgery: A Meta-Analysis. Arch. Surg..

[19] Karcz M., Papadakos P.J. (2013). Respiratory Complications in the Postanesthesia Care Unit: A Review of Pathophysiological Mechanisms. Can. J. Respir. Ther. CJRT Rev. Can. Ther. Respir. RCTR.

[20] Shah A.C., Nair B.G., Spiekerman C.F., Bollag L.A. (2017). Continuous Intraoperative Epidural Infusions Affect Recovery Room Length of Stay and Analgesic Requirements: A Single-Center Observational Study. J. Anesth..

[21] Ganter M.T., Blumenthal S., Dübendorfer S., Brunnschweiler S., Hofer T., Klaghofer R., Zollinger A., Hofer C.K. (2014). The Length of Stay in the Post-Anaesthesia Care Unit Correlates with Pain Intensity, Nausea and Vomiting on Arrival. Perioper. Med..

[22] Chung F., Ritchie E., Su J. (1997). Postoperative Pain in Ambulatory Surgery. Anesth. Analg..

[23] Cohen J.B., Smith B.B., Teeter E.G. (2024). Update on Guidelines and Recommendations for Enhanced Recovery after Thoracic Surgery. Curr. Opin. Anaesthesiol..

[24] Wegener S.T., Castillo R.C., Haythornthwaite J., MacKenzie E.J., Bosse M.J. (2011). LEAP Study Group. Psychological Distress Mediates the Effect of Pain on Function. Pain.

[25] Batchelor T.J.P., Rasburn N.J., Abdelnour-Berchtold E., Brunelli A., Cerfolio R.J., Gonzalez M., Ljungqvist O., Petersen R.H., Popescu W.M., Slinger P.D. (2019). Guidelines for Enhanced Recovery after Lung Surgery: Recommendations of the Enhanced Recovery After Surgery (ERAS^®^) Society and the European Society of Thoracic Surgeons (ESTS). Eur. J. Cardio-Thorac. Surg..

[26] Kehlet H., Jensen T.S., Woolf C.J. (2006). Persistent Postsurgical Pain: Risk Factors and Prevention. Lancet.

[27] Döpfmer U.R., Schenk M.R., Kuscic S., Beck D.H., Döpfmer S., Kox W.J. (2001). A Randomized Controlled Double-Blind Trial Comparing Piritramide and Morphine for Analgesia after Hysterectomy. Eur. J. Anaesthesiol..

[28] Zapletal B., Bsuchner P., Begic M., Slama A., Vierthaler A., Schultz M.J., Tschernko E.M., Wohlrab P. (2025). Effectiveness and Safety of Erector Spinae Plane Block vs. Conventional Pain Treatment Strategies in Thoracic Surgery. J. Clin. Med..

[29] Jneid H., Fonarow G.C., Cannon C.P., Hernandez A.F., Palacios I.F., Maree A.O., Wells Q., Bozkurt B., Labresh K.A., Liang L. (2008). Sex Differences in Medical Care and Early Death After Acute Myocardial Infarction. Circulation.

[30] Fang J., Alderman M.H. (2006). Gender Differences of Revascularization in Patients With Acute Myocardial Infarction. Am. J. Cardiol..

[31] Lopez R., Snair M., Arrigain S., Schold J.D., Hustey F., Walker L.E., Phelan M.P. (2021). Sex-Based Differences in Timely Emergency Department Evaluations for Patients with Drug Poisoning. Public Health.

[32] Sarton E., Olofsen E., Romberg R., den Hartigh J., Kest B., Nieuwenhuijs D., Burm A., Teppema L., Dahan A. (2000). Sex Differences in Morphine Analgesia. Anesthesiology.

[33] Paller C.J., Campbell C.M., Edwards R.R., Dobs A.S. (2009). Sex-Based Differences in Pain Perception and Treatment. Pain Med..

[34] Hoffmann D.E., Fillingim R.B., Veasley C. (2022). The Woman Who Cried Pain: Do Sex-Based Disparities Still Exist in the Experience and Treatment of Pain?. J. Law Med. Ethics A J. Am. Soc. Law Med. Ethics.

[35] Calderone K.L. (1990). The Influence of Gender on the Frequency of Pain and Sedative Medication Administered to Postoperative Patients. Sex Roles.

[36] Hallet J., Sutradhar R., Flexman A., McIsaac D.I., Carrier F.M., Turgeon A.F., McCartney C., Chan W.C., Coburn N., Eskander A. (2024). Association between Anaesthesia–Surgery Team Sex Diversity and Major Morbidity. Br. J. Surg..

[37] Miyawaki A., Jena A.B., Rotenstein L.S., Tsugawa Y. (2024). Comparison of Hospital Mortality and Readmission Rates by Physician and Patient Sex. Ann. Intern. Med..

[38] Heybati K., Chang A., Mohamud H., Satkunasivam R., Coburn N., Salles A., Tsugawa Y., Ikesu R., Saka N., Detsky A.S. (2025). The Association between Physician Sex and Patient Outcomes: A Systematic Review and Meta-Analysis. BMC Health Serv. Res..

[39] Hudcova J., McNicol E.D., Quah C.S., Lau J., Carr D.B. (2006). Patient Controlled Opioid Analgesia versus Conventional Opioid Analgesia for Postoperative Pain. Cochrane Database Syst. Rev..

[40] McNicol E.D., Ferguson M.C., Hudcova J. (2015). Patient Controlled Opioid Analgesia versus Non-patient Controlled Opioid Analgesia for Postoperative Pain. Cochrane Database Syst. Rev..

[41] Bainbridge D., Martin J.E., Cheng D.C. (2006). Patient-Controlled versus Nurse-Controlled Analgesia after Cardiac Surgery—A Meta-Analysis. Can. J. Anaesth..

[42] Zhou Y., Huang J.-X., Lu X.-H., Zhang Y.-F., Zhang W. (2015). Patient-Controlled Intravenous Analgesia for Non-Small Cell Lung Cancer Patient after Thoracotomy. J. Cancer Res. Ther..

[43] Via L.L., Cavaleri M., Terminella A., Sorbello M., Cusumano G. (2024). Loco-Regional Anesthesia for Pain Management in Robotic Thoracic Surgery. J. Clin. Med..

