# Supplementary material for: Analgesia and Pain in Female and Male Patients After Video-Assisted Thoracic Surgery: A Study Under Real-World Conditions
Source: J Clin Med. 2026 Feb 10;15(4):1397. doi: 10.3390/jcm15041397 (PMC12942130; doi:10.3390/jcm15041397)
Supplement: Supplementary file 1 [file jcm-15-01397-s001.zip › Table S2.pdf]

Table S2. NRS and Piritramide Dose in first 6 Hours after Arrival in the PACU

	female patients (N = 11)	male patients (N = 13)	*P*
NRS			
Median NRS in first 6 hours [IQR]	2.5 [1.3 to 4.3]	2.8 [1.6 to 3.5]	0.88
Piritramide dose			
Median piritramide dose in first 6 hours, mg [IQR]	12.0 [7.5 to 24.0]	9.5 [6.8 to 18.8]	0.65

Data are presented as medians with interquartile ranges [IQR]; Abbreviations: NRS, numerical rating scale; PACU, Post–anesthesia Care Unit;
