# Supplementary material for: Analgesia and Pain in Female and Male Patients After Video-Assisted Thoracic Surgery: A Study Under Real-World Conditions
Source: J Clin Med. 2026 Feb 10;15(4):1397. doi: 10.3390/jcm15041397 (PMC12942130; doi:10.3390/jcm15041397)
Supplement: Supplementary file 1 [file jcm-15-01397-s001.zip › Table S1.pdf]

Table S1. **Adverse events, LOS in the PACU**

	all female teams AFT (N = 33)	all male teams AMT (N = 19)	mixed teams MT (N = 148)	P
All patients				
analgesia failure	1 (3.0)	1 (5.3)	11 (7.4)	0.63
regional anesthesia related AE	0 (0.0)	0 (0.0)	0 (0.0)	-
PONV	8 (24.2)	4 (21.1)	35 (23.6)	0.96
delirium	0 (0.0)	0 (0.0)	1 (0.7)	0.84
LOS in the PACU (min)	135.0 [109.0 to 180.0]	120.0 [90.0 to 245.0]	125.0 [108.3 to 180.0]	0.85
Female patients				
analgesia failure	1 (5.6)	1 (10.0)	6 (9.4)	0.87
regional anesthesia related AE	0 (0.0)	0 (0.0)	0 (0.0)	-
PONV	6 (33.3)	3 (30.0)	21 (32.8)	0.98
delirium	0 (0.0)	0 (0.0)	0 (0.0)	-
LOS in the PACU (min)	127.5 [108.0 to 168.7]	120.0 [101.3 to 401.3]	120.0 [101.3 to 157.0]	0.93
Male patients				
analgesia failure	0 (0.0)	0 (0.0)	5 (6.0)	0.47
regional anesthesia related AE	0 (0.0)	0 (0.0)	0 (0.0)	-
PONV	2 (13.3)	1 (11.1)	14 (16.7)	0.87
delirium	0 (0.0)	0 (0.0)	1 (1.2)	0.86
LOS in the PACU (min)	140.0 [109.0 to 200.0]	110.0 [90.0 to 247.5]	130.0 [110.0 to 180.0]	0.55
All patients				
analgesia failure	1 (3.0)	1 (5.3)	11 (7.4)	0.63
regional anesthesia related AE	0 (0.0)	0 (0.0)	0 (0.0)	-
PONV	8 (24.2)	4 (21.1)	35 (23.6)	0.96
delirium	0 (0.0)	0 (0.0)	1 (0.7)	0.84
LOS in the PACU (min)	135.0 [109.0 to 180.0]	120.0 [90.0 to 245.0]	125.0 [108.3 to 180.0]	0.85

Data are presented as numbers (%) and medians with interquartile ranges [IQR]; PACU, Post–anesthesia Care Unit; BW, bodyweight; VAS used a scale of 1 to 10. Abbreviations: AE, adverse event; PONV, postoperative nausea and vomiting; LOS, length of stay; PACU, postoperative anesthesia care unit; min, minutes.
