# Supplementary figures and images for: Analgesia and Pain in Female and Male Patients After Video-Assisted Thoracic Surgery: A Study Under Real-World Conditions
Source: J Clin Med. 2026 Feb 10;15(4):1397. doi: 10.3390/jcm15041397 (PMC12942130; doi:10.3390/jcm15041397)

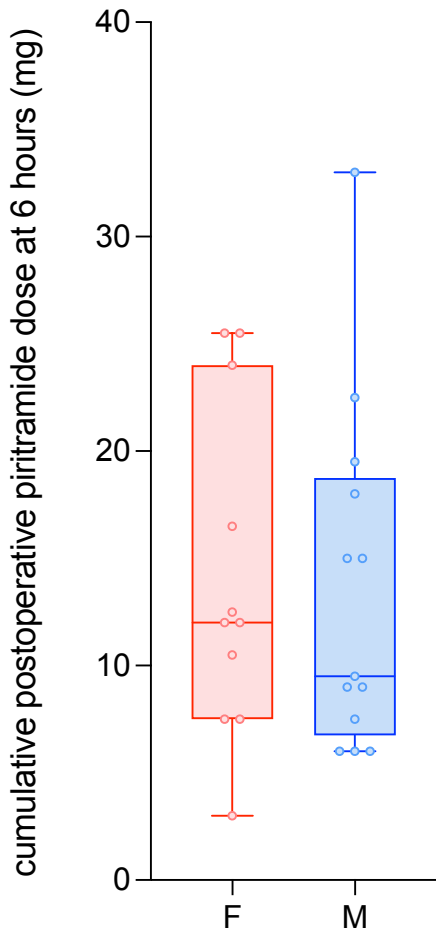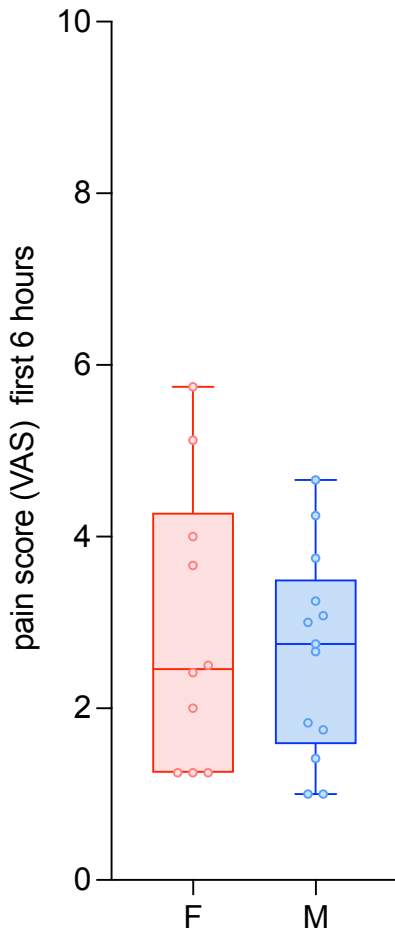

Supplement: Supplementary file 1 [file jcm-15-01397-s001.zip › Figure S1.pdf]
